# Lubricants for the promotion of sexual health and well-being: a systematic review

**DOI:** 10.1080/26410397.2022.2044198

**Published:** 2022-03-22

**Authors:** Caitlin E. Kennedy, Ping Teresa Yeh, Jingjia Li, Lianne Gonsalves, Manjulaa Narasimhan

**Affiliations:** a Associate Professor, Department of International Health, Johns Hopkins Bloomberg School of Public Health, Baltimore, MD, USA. *Correspondence:* caitlinkennedy@jhu.edu; b Research Associate, Department of International Health, Johns Hopkins Bloomberg School of Public Health, Baltimore, MD, USA; c Research Assistant, Department of International Health, Johns Hopkins Bloomberg School of Public Health, Baltimore, MD, USA; d Technical Officer, Department of Antimicrobial Resistance, World Health Organization, Geneva, Switzerland; e Scientist, Department of Sexual and Reproductive Health and Research, World Health Organization, (includes the UNDP/UNFPA/UNICEF/WHO/World Bank Special Programme of Research, Development and Research Training in Human Reproduction – HRP), Geneva, Switzerland

**Keywords:** lubricants, sexual health, systematic review, vaginal dryness, dyspareunia, anal sex

## Abstract

Promoting sexual health is a World Health Organization (WHO) priority. Lubricants are widely available and used to improve sexual pleasure and reduce pain during intercourse. To inform WHO’s self-care interventions guideline, we conducted a systematic review of the peer-reviewed literature to answer the question: does use of lubricants during or prior to sex result in improved sexual health and well-being. We searched PubMed, CINAHL, LILACS and EMBASE on 8 July 2020 for effectiveness, values and preferences, and cost data related to commercially available vaginal and anal lubricants. Data were systematically extracted and qualitatively synthesised. Effectiveness evidence was summarised in GRADE evidence profiles. Seven studies met the effectiveness review criteria. Two randomised trials found lubricant use led to improved female sexual well-being and had no impact on incidence of human papillomavirus (moderate certainty evidence). One observational study with gay and bisexual men showed lubricants were associated with increased reports of pain during receptive intercourse and no difference in pain during insertive intercourse, but a reduced degree of pain in both types of intercourse (low/very low certainty evidence). One observational study with female breast cancer survivors found better outcomes of vaginal dryness and dyspareunia with lubricant use (very low certainty evidence). Twenty-one values and preferences studies from diverse populations globally found that most individuals supported lubricant use for reasons of comfort/reduced pain and sexual pleasure. No cost studies were identified. Although evidence is limited, lubricants appear to offer an acceptable approach to improving sexual health and well-being.

## Résumé

Promouvoir la santé sexuelle est une priorité de l’Organisation mondiale de la santé (OMS). Les lubrifiants sont largement disponibles et utilisés pour améliorer le plaisir sexuel et réduire les douleurs pendant les rapports. Pour guider les principes directeurs de l’OMS sur les interventions d’auto-prise en charge, nous avons examiné systématiquement les publications à comité de lecture afin de répondre à la question: l’utilisation de lubrifiants pendant ou avant les rapports sexuels aboutit-elle à une meilleure santé sexuelle et un bien-être accru? Le 8 juillet 2020, nous avons recherché dans PubMed, CINAHL, LILACS et EMBASE des données sur l’efficacité, les valeurs et préférences, ainsi que sur les coûts, relatives aux lubrifiants vaginaux et anaux disponibles dans le commerce. Les données ont été extraites de manière systématique puis synthétisées qualitativement. Les données sur l’efficacité ont été résumées dans les profils de données de GRADE. Sept études réunissaient les critères de l’examen de l’efficacité. Deux essais randomisés ont montré que l’utilisation de lubrifiants améliorait le bien-être des femmes et n’avait pas d’impact sur l’incidence du papillomavirus humain (certitude modérée des données). Une étude observationnelle portant sur des hommes homosexuels et bisexuels a révélé que les lubrifiants étaient associés à des notifications accrues de douleurs pour le partenaire passif et à aucune différence de douleur pour le partenaire actif, mais à un degré de douleur moindre dans les deux types de rapports (certitude faible/très faible des données). Une étude observationnelle auprès de femmes guéries d’un cancer du sein a trouvé de meilleurs résultats en matière de sécheresse vaginale et de dyspareunie avec l’emploi d’un lubrifiant (très faible certitude des données). Vingt et une études sur les valeurs et les préférences auprès de diverses populations ont montré en général que la plupart des individus soutenaient l’utilisation de lubrifiants pour des raisons de confort/réduction de la douleur et plaisir sexuel. Aucune étude de coût n’a été identifiée. Même si les données sont limitées, les lubrifiants semblent offrir une approche acceptable pour l’amélioration de la santé sexuelle et du bien-être.

## Resumen

La promoción de la salud sexual es una prioridad de la Organización Mundial de la Salud (OMS). Los lubricantes están ampliamente disponibles y son utilizados de manera extendida para mejorar el placer sexual y disminuir el dolor durante el coito sexual. Con el fin de informar la guía de la OMS sobre intervenciones en autocuidado, realizamos una revisión sistemática de la literatura revisada por pares para contestar la pregunta ¿el uso de lubricantes durante o antes del sexo contribuye al mejoramiento de la salud y el bienestar sexuales? El 8 de julio de 2020, realizamos una búsqueda en PubMed, CINAHL, LILACS y EMBASE de eficacia, valores y preferencias, y datos de costo relacionados con lubricantes vaginales y anales disponibles comercialmente. Los datos fueron extraídos sistemáticamente y sintetizados cualitativamente. La evidencia sobre la eficacia se resumió en perfiles de evidencia GRADE. Siete estudios reunieron los criterios de la revisión de eficacia. Dos ensayos clínicos aleatorizados encontraron que el uso de lubricantes contribuye al mejoramiento del bienestar sexual de las mujeres y que no tuvo ningún impacto en la incidencia del virus del papiloma humano (certeza de evidencia moderada). Un estudio observacional con hombres homosexuales y bisexuales mostró que los lubricantes estaban asociados con un mayor número de informes de dolor durante el coito receptivo y ninguna diferencia en dolor durante el coito insertivo, pero con menor grado de dolor en ambos tipos de coito (certeza de evidencia baja o muy baja). Un estudio observacional con sobrevivientes de cáncer de mama encontró mejores resultados del uso de lubricantes con relación a la sequedad vaginal y la dispareunia (certeza de evidencia muy baja). En 21 estudios de valores y preferencias de diversas poblaciones mundialmente, se encontró que la mayoría de las personas apoyan el uso de lubricantes por razones de comodidad/disminución del dolor y placer sexual. No se identificó ningún estudio de costo. Aunque existe evidencia limitada, al parecer, los lubricantes ofrecen un enfoque aceptable para mejorar la salud y el bienestar sexuales.

## Introduction

Promoting sexual health is one of the five priority areas of the World Health Organization (WHO) reproductive health strategy.^1^ WHO’s working definition of sexual health is the “state of physical, emotional, mental and social well-being in relation to sexuality; it is not merely the absence of disease, dysfunction or infirmity. Sexual health requires a positive and respectful approach to sexuality and sexual relationships, as well as the possibility of having pleasurable and safe sexual experiences, free of coercion, discrimination and violence”.^2^

Use of lubricants during sex may result in improved sexual health and well-being and may be particularly helpful for individuals experiencing vaginal dryness associated with menopause,^3^ individuals experiencing dyspareunia (pain during sexual intercourse or other sexual activity that involves vaginal penetration),^4^ or people engaging in anal sex.^5^ Lubricants may also facilitate optimal sexual function, pleasure, and enjoyment for sexually active individuals, across genders, regardless of specific health conditions, and improve sexual relationships. Lubricants have also been recommended for use in conjunction with condoms to reduce condom breakage and therefore provide better protection against sexually transmitted infections (STIs), including HIV.^6^ There is a wide range of lubricant products available on the market globally, which are used for both anal and vaginal sexual activity. However, while lubricant use may be generally helpful, substandard products used as lubricants could also result in adverse health outcomes.

We sought to systematically review the evidence for the use of lubricants during or prior to sex to improve sexual health and well-being. We conducted this review in the context of expanding the evidence base of WHO’s normative guidance on self-care interventions^7^ to interventions that promote sexual health. Lubricants can be used in isolation or with other products, such as condoms, but are generally over-the-counter products used by individuals and their partners without prescription or involvement of health workers; they are thus a form of self-care, which WHO defines as “the ability of individuals, families and communities to promote health, prevent disease, maintain health and cope with illness and disability with or without the support of a health worker”.^7^ Current WHO guidance regarding self-care interventions and lubricants includes a good practice statement that “people from underserved populations should be able to experience full, pleasurable sex lives and have access to a range and choice of reproductive health options”^7^ and an advisory note recommending procurement of additional lubricants for male and female condoms, with specific considerations for certain lubricant characteristics, such as osmolality and pH.^6^ This review was also conducted as part of a response to the COVID-19 pandemic during which self-care interventions for sexual and reproductive health have been prioritised.^8^ Finally, this review supports the move to improve universal health coverage for all, as implementation of self-care interventions within the context of human rights, gender equality, and a life course approach can promote comprehensive, integrated, and people-centred approaches to health service delivery.^9^

## Methods

This review addressed the question: Does use of lubricants during or prior to sex result in improved sexual health and well-being? We reviewed the extant literature in three areas relevant to answering these questions and developing WHO guidance: the effectiveness of the intervention, the values and preferences of end users and health workers, and cost information. The review followed PRISMA guidelines,^10^ and the protocol was published on PROSPERO (registration number CRD42020208976).

For the purposes of this review, we focused on vaginal and anal lubricants (used immediately prior to or during sexual activity) as opposed to vaginal moisturisers (used daily over a longer term). A recent review^11^ describes the difference between vaginal lubricants and vaginal moisturisers as follows:

“Lubricants may relieve vaginal dryness and discomfort during sexual activity, providing short-term relief from vaginal dryness and dyspareunia. Vaginal moisturisers are intended to be used primarily for the relief of vaginal dryness on a day-to-day basis, to provide comfort and offer long-term benefits. Vaginal moisturisers are classified as Class IIa Medical Devices by the Medicines and Healthcare products Regulatory Agency, based on the intended duration of their use (vaginal moisturisers are intended to be present in the body for longer than 60 min, but a single application should not last longer than 30 days). Lubricants may or may not be classified as medical devices, depending on their individual claims.”

We included products that had no known harmful effects for use as lubricants (e.g. olive oil) but excluded biological lubricants (e.g. saliva, pre-seminal fluid) and microbicide gels. We did not focus on the range of vaginal drying products, bleaching products, or other topical anal or vaginal products that are available across settings.

### Effectiveness review

The effectiveness review was designed according to the PICO format as follows:

**Population:** Sexually active individuals (with attention to specific subpopulations in the stratifications noted below)

**Intervention:** Use of lubricant during sexual activity (defined as any penetration, including vaginal/anal, with/without a partner, and with an object)

**Comparison:** Sexual activity without lubricant

**Outcomes:**

1. Vaginal dryness or pain during vaginal/anal penetration.
2. Sexual arousal dysfunctions (female sexual arousal dysfunction, male erectile dysfunction).
3. Sexual desire, arousal, lubrication, orgasm, satisfaction, and pleasure.
4. Vaginal discharge and bacterial vaginosis.
5. Side effects (irritation, infections [yeast; reproductive tract infection (RTI); STIs; urinary tract infection (UTI)]).
6. STIs/HIV (incidence, prevalence, transmission, etc.).
7. Self-efficacy, self-determination, autonomy, and empowerment around sexual health, and sexuality (confidence, communication with partners, self-esteem).
8. Other side effects or adverse events, or social harms (e.g. coercion, violence [including intimate-partner violence, violence from family members or community members, etc.], psycho-social harm, self-harm, etc.), and whether these harms were corrected/had redress available.

To be included in the review, an article had to meet the following criteria:

1. A study design that compared self-use of lubricants during sexual activity to sexual activity without lubricants. Comparative study designs included randomised controlled trials, non-randomised analytical trials, and comparative observational studies (including prospective analytical cohort studies, cross-sectional studies, analytical before-after studies and interrupted time series) that compared individuals who received the intervention to those who did not.
2. Measured one or more of the outcomes listed above.
3. Published in a peer-reviewed journal.

No restrictions were placed based on location of the intervention. No language restrictions were used on the search. Articles in English, French, Spanish, and Chinese were coded directly; articles in other languages were translated. No restrictions were placed on the date of publication, other than the search cut-off date.

The search strategy, designed for PubMed and adapted for other databases, combined search terms for two concepts: lubricants and sex (see Supplementary material). These search terms were used both for the main systematic review (PICO question) and for the values and preferences and cost reviews (described below).

The following electronic databases were searched through the search date of July 8, 2020: PubMed, CINAHL, LILACS, and EMBASE. Secondary reference searching was also conducted on all studies included in the review. We searched for ongoing randomised controlled trials (RCTs) through clinicaltrials.gov, the WHO International Clinical Trials Registry Platform, the Pan-African Clinical Trials Registry, and the Australian New Zealand Clinical Trials Registry. We also conducted a handsearch on Google Scholar and the Cochrane Library. Finally, selected experts in the field were contacted to identify additional articles not identified through other search methods.

Titles, abstracts, citation information, and descriptor terms of citations identified through the search strategy were screened by a member of the study staff. Full-text articles were obtained of all selected abstracts, and two independent reviewers assessed all full-text articles for eligibility to determine final study selection. Differences were resolved through consensus. Data were extracted independently by two reviewers using standardised data extraction forms. Differences in data extraction were resolved through consensus and referral to a senior study team member from WHO when necessary.

The following information was gathered from each included study:

- Study identification: author(s); type of citation; year of publication.
- Study description: study objectives; location; population characteristics; type of lubricant; study design; sample size; follow-up periods and loss to follow-up.
- Outcomes: analytic approach; outcome measures; comparison groups; effect sizes; confidence intervals; significance levels; conclusions; limitations.

For randomised trials, risk of bias was assessed using the Cochrane Collaboration’s tool for assessing risk of bias.^12^ For non-randomised trials but comparative studies, risk of bias was assessed using the Evidence Project 8-item checklist for intervention evaluations.^13^

Data were analysed according to coding categories and outcomes. If we had identified multiple studies reporting the same outcome measured in the same way, meta-analysis would have been conducted using random-effects models to combine risk ratios with Comprehensive Meta-Analysis (CMA).

We planned to stratify all analyses by the following categories, where data were available:

- Condom use
- Point of access (e.g. stores, pharmacies, online/telehealth, etc.)
- Type of lubricant
- Frequency of sex
- Populations (e.g. adults/adolescents, individuals with specific medical conditions or on specific medications, perimenopausal/menopausal persons, persons with disabilities, postpartum, sex workers, sexual and gender minorities, race/ethnicity, etc.)
- Vaginal vs. anal sex
- Type of partner (transactional or not, steady vs. casual)
- Vulnerabilities (e.g. poverty, disability, literacy/educational level)
- High-income versus low or middle-income countries

Data were summarised in GRADE Evidence Profile tables using GRADEPro.

### Values and preferences review

Values and preferences have been defined as the “collection of goals, expectations, predispositions, and beliefs that individuals have for certain decisions and their potential outcomes”^14^ and are a required part of the WHO guideline development process.^15^ The same search terms were used to search and screen for studies to be included in the values and preferences review. Studies were included in this review if they presented primary data examining preferences of lubricant users, or individuals who might be candidates for lubricant use. We focused on studies examining the values and preferences of end users, but also included studies examining the values and preferences of health workers. We considered issues related to age of availability, informed decision-making, coercion, and seeking redress in this section. These studies could be qualitative or quantitative in nature, but had to present primary data collection – think pieces and review articles were not be included. Values and preferences literature was summarised qualitatively and was organised by study design and methodology, location, and population.

### Cost review

Consideration of costs and resource use is also a required component of the WHO guideline development process.^15^ The same search terms were used to search and screen for studies to be included in the cost review. Studies were included in this review if they presented primary data comparing costing, cost-effectiveness, cost-utility, or cost-benefit of the intervention and comparison listed in the PICO above, or if they presented cost-effectiveness of the intervention as it relates to the PICO outcomes listed above. We planned to summarise cost literature qualitatively. We planned to classify cost literature into four categories (health sector costs, other sector costs, patient/family costs, and productivity impacts) and within each category organise by study design/methodology, location, and population.

## Results

**Figure 1.**
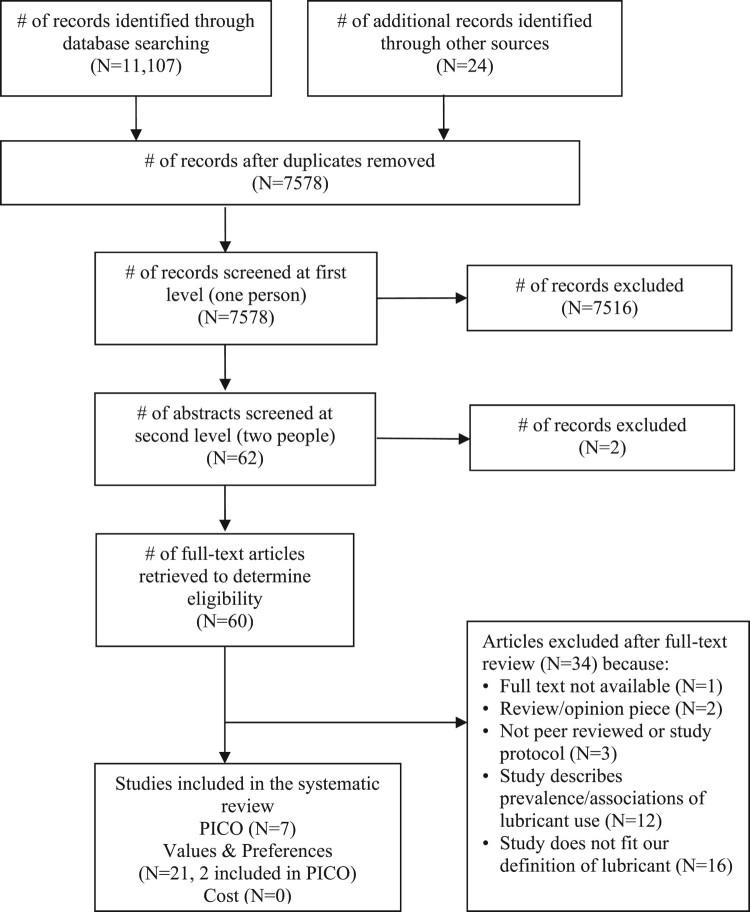
PRISMA flow chart showing disposition of citations through the search and screening process.

Our search yielded 7578 unique references, of which 60 were retained for full-text review (Figure 1). Ultimately, we identified seven that met the inclusion criteria for the effectiveness review,^4,16–21^ twenty-one values and preferences studies,^4,17,22–40^ and no cost studies. A table of excluded studies is provided in Supplementary Table A.

### Effectiveness review

Overall, seven studies met the inclusion criteria for the effectiveness review.^4,16–21^ This included two RCTs and five observational studies. Table 1 presents descriptive data from the two RCTs and the two observational studies presented in the GRADE evidence profile^4,17,20,21^; we included RCT data in GRADE for each outcome category when available, and where RCT data were not available, we included data from observational studies. Table 2 presents a GRADE Evidence Profile for these four studies. Overall, the quality of evidence for each outcome rated from moderate (three outcomes) to very low (six outcomes) in the GRADE system. Given the small number of studies presenting outcome data, no further stratifications from our *a priori* list were possible, and meta-analysis was not conducted. Table 3 provides a summary of findings from all seven included studies, which are described in the text below.

**Table 1.** Description of studies included in GRADE.

Study	Location	Population	Sampling and Study design	Intervention	Comparator	Outcomes
**RCTs**						
**Rosen et al., 2010**^20^	USA: 3 cities	Sexually active adult women in a stable heterosexual partnership (*n* = 326; mean (SD) age = 44 (13); age range 21–77; 78% White, 15% African American, 5% Hispanic, 2% Other)	Non-probability (facility-based) RCT	1. Couple lubricants (one lubricant for use by the woman and a second lubricant for concurrent use by her male partner) (*n* = 80, mean age = 44) 2. Female lubricant (lubricant for use by the woman) (*n* = 82, mean age = 44)	No lubricant use (*n* = 82, mean age = 44)	*3. Sexual desire, arousal, lubrication, orgasm, satisfaction, and pleasure:* Female sexual well-being scale and sub-domains
**Sawaya et al., 2008**^21^	Zimbabwe: Chitungwiza and Epworth (outside Harare)	HIV-negative adult women (*n* = 2040; no ethnicity data reported)	Non-probability (facility-based) RCT	Received a clinician-fitted latex diaphragm (All-Flex® Arcing Spring diaphragm), a supply of lubricant gel (Replens^TM^), and male condoms (*n* = 1020; age ≤24, 38.3%; 25–34, 45.6%; ≥35, 16.1%)	No lubricant use / condom use only (*n* = 1020; age ≤24, 36.0%; 25–34, 46.3%; ≥35, 17.8%.)	*6. STIs/HIV:* Incidence of any HPV type and oncogenic HPV types
**Observational studies**						
**Dodge et al., 2015**^17^	USA (internet)	Self-identified gay (*n* = 307) and bisexual (*n* = 25) adult participants in the 2012 National Survey of Sexual Health and Behaviour (NSSHB) who reported sexual behaviours with other male partners during their last sexual encounter (*n* = 333; age range: 18–70+; 68.4% White, 4.2% Black, 6.6 Other, 18.5% Hispanic, 2.3% more than two races)	Non-probability (convenience) Cross-sectional survey	Used commercial lubricant product(s) at last sexual event (*n* = 163)	No lubricant use (*n* = 170)	*Vaginal dryness or pain during vaginal/anal penetration:* Dryness/pain during penetration
**Juraskova et al., 2013**^4^	Australia	Adult breast cancer survivors in a sexual relationship (*n* = 25; mean age at diagnosis = 47; current age mean = 51, current age range:37–66; 76% have children)	Non-probability (convenience) Before-after study	Used lubricant (olive oil, pelvic floor muscle relaxation exercises, vaginal moisturiser) (*n* = 25)	No lubricant use (*n* = 25, same participants but before using lubricants)	*1. Vaginal dryness or pain during vaginal/anal penetration:* Dyspareunia, Sexual discomfort

**Table 2.** GRADE Evidence Profile.

Certainty assessment							№ of patients		Effect		Certainty	Importance
№ of studies	Study design	Risk of bias	Inconsistency	Indirectness	Imprecision	Other considerations	lubricants during or prior to sex	no lubricants	Relative (95% CI)	Absolute (95% CI)		
***1. Vaginal dryness or pain during vaginal/anal penetration:*Experience of pain during last insertive partnered sexual event (assessed with: self-report)**												
1^17^	Observational studies	Serious^a^	Not serious^b^	Not serious	Serious^c^	None	11/61 (18.0%)	3/21 (14.3%)	**RR 1.26** (0.39–4.09)	**37 more per 1000** (from 87 fewer to 441 more)	⊕◯◯◯ VERY LOW	CRITICAL
***1. Vaginal dryness or pain during vaginal/anal penetration:*Experience of pain during last receptive partnered sexual event (assessed with: self-report)**												
1^17^	Observational studies	Very serious^a,d^	Not serious^b^	Not serious	Not serious	None	45/71 (63.4%)	3/17 (17.6%)	**RR 3.59** (1.27–10.18)	**457 more per 1000** (from 48 more to 1000 more)	⊕◯◯◯ VERY LOW	CRITICAL
***1. Vaginal dryness or pain during vaginal/anal penetration:*Degree of pain during last insertive partnered sexual event (assessed with: self-report, higher score indicates greater pain)**												
1^17^	Observational studies	Serious^a^	Not serious^b^	Not serious	Not serious	None	2.3	2.9	MD **0.6 lower**		⊕◯◯◯ VERY LOW	CRITICAL
***1. Vaginal dryness or pain during vaginal/anal penetration:* Degree of pain during last receptive partnered sexual event (assessed with: self-report, higher score indicates greater pain)**												
1^17^	Observational studies	Serious^a^	Not serious^b^	Not serious	Not serious	None	2.2	3	MD **0.8 lower**		⊕◯◯◯ VERY LOW	CRITICAL
***1. Vaginal dryness or pain during vaginal/anal penetration:*Dyspareunia (assessed with: visual analogue score pain assessment of dyspareunia, lower score is better outcome; Scale from: 0–10)**												
1^4^	Observational studies	Serious^a^	Not serious^b^	Not serious	Serious^e^	None	2.7	7	MD **4.3 lower**		⊕◯◯◯ VERY LOW	CRITICAL
***1. Vaginal dryness or pain during vaginal/anal penetration:*Sexual discomfort (assessed with: Sexual Activity Questionnaire – Discomfort subscale assessing vaginal dryness and dyspareunia, higher score is better outcome; Scale from: 0–6)**												
1^4^	Observational studies	Serious^a^	Not serious^b^	Not serious	Serious^e^	None	2.9	0.8	MD **2.1 higher**		⊕◯◯◯ VERY LOW	CRITICAL
***3. Sexual desire, arousal, lubrication, orgasm, satisfaction, and pleasure:*Female sexual well-being (assessed with: FSWB scale overall score)**												
1^20^	Randomised trials	Serious^f^	Not serious^b^	Not serious	Not serious	None	Least-squares mean change in score from baseline vs end of study: Couple lubricant (*n* = 80) vs no lubricant (*n* = 82): 6.35 vs 1.94; Female lubricant (*n* = 82) vs no lubricant (*n* = 82): 3.99 vs 1.94^g^				⊕⊕⊕⃝ MODERATE	IMPORTANT
***6. STIs/HIV:*HPV incidence (one or more new HPV type(s) detected, among participants with no HPV detected at baseline) (follow up: mean 12 months; assessed with: PCR for HPV consensus probe)**												
1^21^	Randomised trials	Not serious^h^	Not serious^b^	Not serious	Serious^c^	None	120/593 (20.2%)	131/587 (22.3%)	**RR 0.91** (0.73–1.13)	**20 fewer per 1000** (from 60 fewer to 29 more)	⊕⊕⊕⃝ MODERATE	IMPORTANT
***6. STIs/HIV:*HPV incidence (one or more new oncogenic HPV type(s) detected, among participants with no HPV detected at baseline) (follow up: mean 12 months; assessed with: PCR for HPV consensus probe)**												
1^21^	Randomised trials	Not serious^h^	Not serious^b^	Not serious	Serious^c^	None	56/593 (9.4%)	51/587 (8.7%)	**RR 1.09** (0.76–1.56)	**8 more per 1000** (from 21 fewer to 49 more)	⊕⊕⊕⃝ MODERATE	IMPORTANT

Notes: CI: Confidence interval; RR: Risk ratio; MD: Mean difference; FSWB: Female sexual well-being; PCR: Polymerase chain reaction; HPV: Human papillomavirus.

^a^ Risk of bias: Certainty of evidence downgraded for self-report of pain.

^b^ Inconsistency: This could not be evaluated, as there is only a single study.

^c^ Imprecision: Certainty of evidence downgraded because 95% CI for RR includes both 1 (no effect) AND either appreciable harm (0.75) or appreciable benefit (1.25).

^d^ Risk of bias: Participants who reported using lubricant during their last partnered event were asked to indicate their reasons for using lubricant. The most highly endorsed statement (89.3%) was that lubricant reduced their pain/discomfort. This may indicate reverse causation between lubricant use and experience of pain.

^e^ Imprecision: Certainty of evidence downgraded due to small sample size (*n* = 25).

^f^ Risk of bias: Certainty of evidence downgraded for detection bias. Blinding was not possible given the nature of the intervention, and outcome may have been affected by lack of blinding.

^g^ FSWB scale includes subscales, whose results (least-squares mean change in score from baseline vs end of study) are presented in this footnote.

**Interpersonal domain:** Couple lubricant (*n* = 80) vs no lubricant (*n* = 82): 1.80 vs 0.13; Female lubricant (*n* = 82) vs no lubricant (*n* = 82): 1.32 vs 0.13.

**Cognitive-emotional domain:** Couple lubricant (*n* = 80) vs no lubricant (*n* = 82): 2.48 vs 1.08; Female lubricant (*n* = 82) vs no lubricant (*n* = 82): 1.67 vs 1.08.

**Physical arousal domain:** Couple lubricant (*n* = 80) vs no lubricant (*n* = 82): 0.81 vs 0.72; Female lubricant (*n* = 82) vs no lubricant (*n* = 82): 0.07 vs 0.72.

**Orgasm satisfaction domain:** Couple lubricant (*n* = 80) vs no lubricant (*n* = 82): 1.43 vs 0.01; Female lubricant (*n* = 82) vs no lubricant (*n* = 82): 1.04 vs 0.01.

^h^ Risk of bias: Certainty of evidence not downgraded for detection bias. Blinding was not possible given the nature of the intervention, but outcome unlikely to have been affected by lack of blinding.

**Table 3.** Comparative findings from studies included in the effectiveness review

Study	Findings
RCTs	
Rosen 2010^20^	*3. Sexual desire, arousal, lubrication, orgasm, satisfaction, and pleasure* Female sexual well-being scale: Least-squares mean change in score from baseline vs end of study: Couple lubricant (*n* = 80) vs no lubricant (*n* = 82): 6.35 vs 1.94; Female lubricant (*n* = 82) vs no lubricant (*n* = 82): 3.99 vs 1.94 Sub-domains of the Female sexual well-being scale were also reported: Interpersonal domain: Couple lubricant (*n* = 80) vs no lubricant (*n* = 82): 1.80 vs 0.13; Female lubricant (*n* = 82) vs no lubricant (*n* = 82): 1.32 vs 0.13. Cognitive-emotional domain: Couple lubricant (*n* = 80) vs no lubricant (*n* = 82): 2.48 vs 1.08; Female lubricant (*n* = 82) vs no lubricant (*n* = 82): 1.67 vs 1.08. Physical arousal domain: Couple lubricant (*n* = 80) vs no lubricant (*n* = 82): 0.81 vs 0.72; Female lubricant (*n* = 82) vs no lubricant (*n* = 82): 0.07 vs 0.72. Orgasm satisfaction domain: Couple lubricant (*n* = 80) vs no lubricant (*n* = 82): 1.43 vs 0.01; Female lubricant (*n* = 82) vs no lubricant (*n* = 82): 1.04 vs 0.01.
Sawaya 2008^21^	*6. STIs/HIV* Incidence of one or more HPV type: Lubricant: 120/593 (20.2%) vs No lubricant: 131/587 (22.3%); Relative Risk (95% CI): 0.91 (0.73–1.13) Incidence of one or more oncogenic HPV types: Lubricant: 56/593 (9.4%) vs No lubricant: 51/587 (8.7%); Relative Risk (95% CI): 1.09 (0.76–1.56)
Observational studies	
Gorbach 2011^18^	*6. STIs/HIV* Rectal STI prevalence: Consistent lubricant use: 9.5% vs Sometimes lubricant use: 2.4% vs Never lubricant use: 4.1% (*p* = 0.019 Fisher exact test)
Juraskova 2013^4^	*1. Vaginal dryness or pain during vaginal/anal penetration* Dyspareunia (lower score is better outcome, scale from 0 to 10): Lubricant: 2.7 (SD = 2.31) vs No lubricant: 7 (SD = 2.40) Sexual discomfort (higher score is better outcome, scale from 0 to 6): Lubricant: 2.9 (SD = 2.05) vs No lubricant: 0.8 (SD = 1.00)
Maierhofer 2016^19^	*6. STIs/HIV* Rectal gonococcal prevalence: comparing lubricant vs. no lubricant, Gun Oil: adjPR 1.99 (95% CI, 1.04–3.80), Slick: adjPR: 3.55 (95% CI, 1.38–9.12); other lubricants (i.e. Wet, KY Jelly, Vaseline, Baby Oil) had no statistically significant associations Rectal chlamydial prevalence: no lubricants (i.e. Gun Oil, Slick, Wet, KY Jelly, Vaseline, Baby Oil) had no statistically significant associations Prevalence of either rectal gonorrhea or rectal chlamydia: comparing lubricant vs. no lubricant, precum: aPR, 1.68 (95%CI, 1.06–2.66), Vaseline: aPR, 1.70 (95% CI, 1.10–2.64), and baby oil: aPR, 2.26 (95% CI, 1.43–3.57) other lubricants had no statistically significant associations
Blair 2020^16^	*6. STIs/HIV* STI (positive test for infectious syphilis and/or rectal gonorrhea and/or rectal chlamydia): Consistent lubricant use during receptive anal intercourse in the last month: 61/91 (67%) vs Never/inconsistent lubricant use: 243/461 (53%), *p* = 0.012.unadjusted OR: 1.81 (95% CI: 1.12–2.93) *p* = 0.015; adjusted OR: 1.81 (95% CI: 1.11–2.96), *p* = 0.018
Dodge 2015^17^	1. *Vaginal dryness or pain during vaginal/anal penetration* Experience of pain during last insertive partnered sexual event: Lubricant: 11/61 (18%) vs No lubricant: 3/21 (14.3%); AOR (CI):0.94 (0.19–4.59) Experience of pain during last receptive partnered sexual event: Lubricant: 45/71 (63.4%) vs No lubricant: 3/17 (17.6%); AOR (CI): 6.25 (1.72–22.75), *p* < 0.005 Degree of pain during last insertive partnered sexual event (higher score indicates greater pain): Lubricant: 2.3 vs No lubricant: 2.9; F = 0.5 Degree of pain during last receptive partnered sexual event (higher score indicates greater pain): Lubricant: 2.2 vs No lubricant: 3.0; F = 14.3, *p* < 0.001

RCT: randomised controlled trial, STI: sexually transmitted infection, HIV: human immunodeficiency virus, HPV: human papillomavirus, adj: adjusted, PR: prevalence ratio, OR: odds ratio, CI: confidence interval

### Sexual desire, arousal, lubrication, orgasm, satisfaction, and pleasure

One RCT^20^ among sexually active adult women in stable heterosexual partnerships in the United States reported on female sexual well-being. This study was graded as moderate certainty evidence due to risk of bias: blinding participants to lubricant use was not possible given the nature of the intervention, and participant reports of sexual well-being may have been affected by their knowledge of lubricant use. Findings indicated that lubricant use was associated with improved female sexual well-being (FSWB scale overall score: Couple lubricant vs no lubricant: 6.35 vs 1.94; Female lubricant vs no lubricant: 3.99 vs 1.94).

### STIs/HIV

One large RCT^21^ among sexually active women in Zimbabwe measured HPV outcomes. This study was graded as moderate certainty evidence due to imprecision, as the 95% confidence interval (CI) for relative risk (RR) crossed 1 and included the potential for both appreciable benefit and appreciable harm. This trial found that lubricant use did not affect the incidence of HPV (any HPV: RR: 0.91, 95% CI: 0.73–1.13; any oncogenic HPV: RR: 1.09, 95% CI: 0.76–1.56).

### Vaginal dryness or pain during vaginal/anal penetration

One observational study^17^ reported the relationship between lubricant use and pain among self-identified gay and bisexual men in the United States. This study was graded as very low certainty of evidence due to potential self-report bias, reverse causality, and imprecision, as the 95% CI crossed 1 and included both appreciable benefit and harm. Pain was assessed through several questions, including whether pain was experienced (yes/no) and degree of pain. Using lubricants was not associated with self-reported pain at last insertive sex (RR: 1.26, 95% CI: 0.39–4.09), but men using lubricants were more likely to report experiencing pain during their last receptive partnered sexual event (RR: 3.59, 95% CI: 1.27–10.18). However, lubricant use was associated with a lower degree of pain reported during both insertive and receptive sex (mean difference: 0.6 lower for insertive sex; 0.8 lower for receptive sex).

A second, small observational study among female breast cancer survivors in Australia^4^ was graded very low certainty evidence due to potential self-report bias and imprecision due to a very small sample size (*n* = 25). This study found lubricant use (specifically olive oil, along with pelvic floor muscle relaxation exercises and vaginal moisturiser) was associated with lower dyspareunia scores (mean difference: 4.3 lower) and lower sexual discomfort scores (mean difference: 2.1 higher in comfort score).

### Other outcomes of interest

No quantitative comparative data were identified from either RCTs or from observational studies related to sexual arousal dysfunctions, vaginal discharge, and bacterial vaginosis, side effects like irritation or infections (yeast, RTI, UTI), and other side effects, adverse events, or social harms.

### Values and preferences review

**Figure 2.**
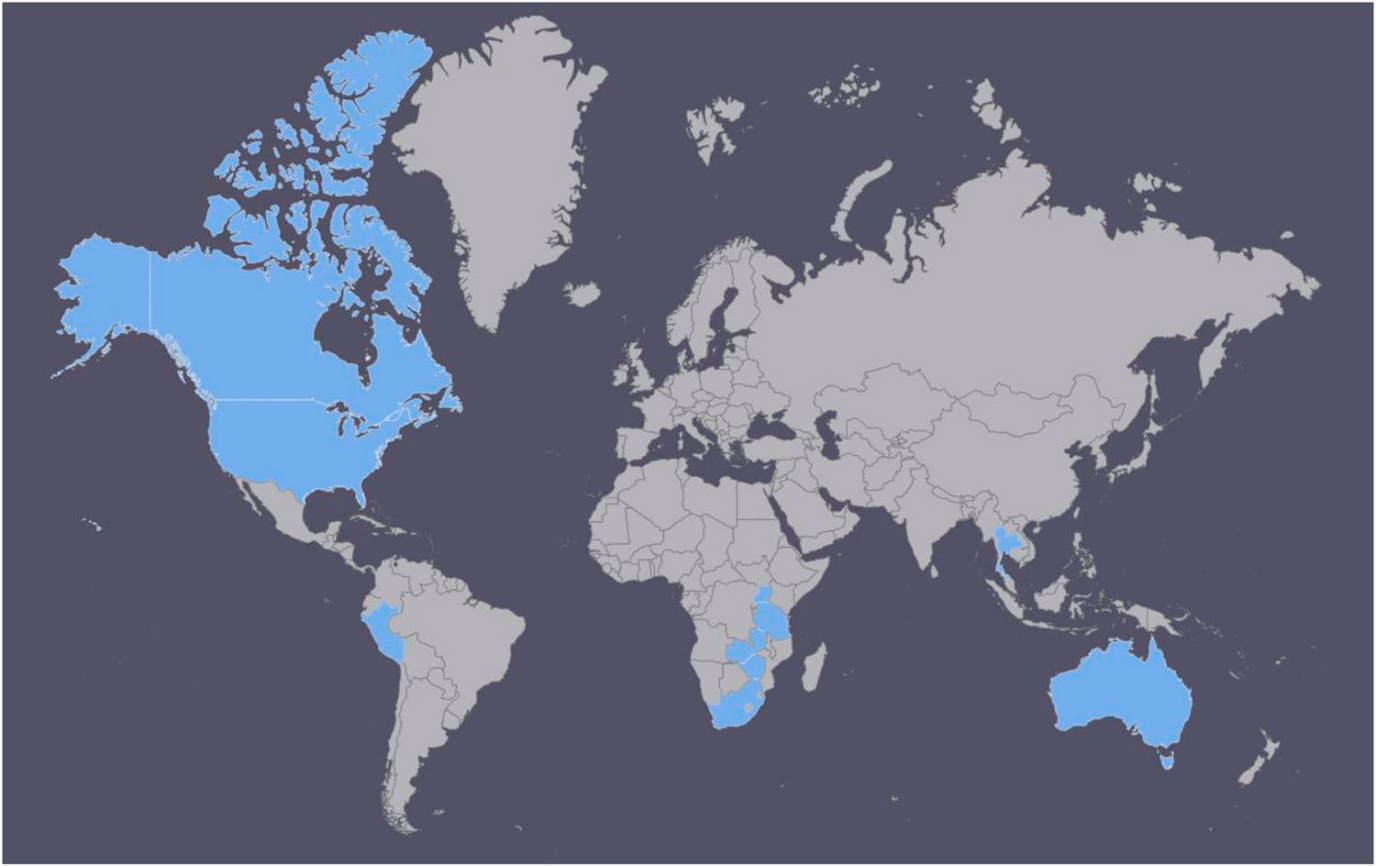
Map showing distribution of studies included in the values and preferences review.

Overall, 21 studies were included in the values and preferences review (Table 4).^4,17,22–40^ The studies were primarily quantitative (*n* = 16, 9 of which were cross-sectional), although there were several qualitative studies (*n* = 4) and a multi-method study (*n* = 1). Twelve were conducted in high-income countries, but others took place in upper-middle (*n* = 6), lower-middle (*n* = 5), and low-income (*n* = 1) countries. Figure 2 presents a map showing the distribution of values and preferences studies globally. The country with the most studies was the USA (*n* = 9), followed by South Africa (*n* = 4), Zimbabwe (*n* = 3), and Australia (*n* = 2). One study each was conducted in Canada, Peru, Tanzania, Thailand, Uganda, and Zambia. One global internet survey was conducted with respondents primarily from North America (also from Europe, Latin America/Caribbean, Asia, Oceania, and other regions). Populations also varied widely, including heterosexuals, men who have sex with men, HIV-infected and HIV-uninfected individuals, individuals with dyspareunia, and clients of STI services.

**Table 4.** Description of studies included in the values and preferences review^1^

Study	Location	Population Description	Study design	Sample size (N)	Key results
Carballo-Diéguez 2000^22^	USA: New York City	318 Latino MSM; 307 were given a survey about sexual practices and 11 participated in a focus group regarding a theoretical microbicidal anal gel	Multi method: structured survey and focus group	318	Participants did not care about the flavour, smell, or colour of a theoretical microbicidal lubricant, but were concerned about how long it would be effective (if applied prior to sex) and the dose needed for effectiveness without interfering with sexual pleasure.
Clark 2013^23^	Peru: Lima	560 MSM recruited from Lima STI and HIV testing sites or through STI screening outreach programmes	Qualitative: in depth individual and group interviews	560	MSM engaged in receptive sex preferred using lubricants for sex more than those engaged in insertive sex. Some preferred the pain of “dry” sex because of their partner’s pleasure. MSM who also had sex with women did not find it as acceptable to use lubricant with female partners and primarily did so with male partners. Most participants preferred commercial lubricants provided by local pharmacies or clinics over substitutes such as saliva, body fluids, or household products but would use these alternatives if commercial lubricant was not readily available.
Dodge 2015^17^	USA: national	Self-identified gay (*n* = 307) and bisexual (*n* = 25) adult participants in the 2012 National Survey of Sexual Health and Behaviour (NSSHB) who reported sexual behaviours with other male partners during their last sexual encounter	Quantitative: cross-sectional study	712	Primary reasons for using lubricants were to make sex more comfortable (68.8%), to reduce pain during sex (49.9%), and to increase pleasure during sex (40.9%). Other reasons included easier/faster/higher-quality orgasms, to make sex more wet or fun, to enhance foreplay, and to reduce the chances of the condom drying out.
Duby 2016^24^	South Africa, Uganda, Zimbabwe	88 women from South Africa (*n* = 20), Uganda (*n* = 22) and Zimbabwe (*n* = 26), who formerly participated in VOICE, an HIV prevention trial of two antiretroviral oral tablets and a vaginal gel. Mean age = 28.6, age range 20–40	Qualitative: in-depth interviews	88	Lubricants were used for penile-anal intercourse to make sex clean, fast, easy insertion; not used because some (e.g. Vasoline) may degrade latex and lead to condom breakage.
Herbenick 2011^25^	USA	Adult women (*N* = 2453) from the U.S. Mean age = 32.69, age range 18–68; most (85.4%) described their ethnicity as White; most (86.5%) self-identified as heterosexual.	Quantitative: prospective cohort following for 5 weeks with randomisation and double-blinding pre-post design (given lubricants as the intervention)	2453	Among penile – vaginal sex events, participants’ self-reports on measures of sexual pleasure and sexual satisfaction were significantly higher for events that included the use of a water-based lubricant or silicone-based lubricant compared with no lubricant use. For penile – anal events, ratings of sexual pleasure and satisfaction were significantly higher for events associated with water-based lubricant over no lubricant. All lubricant types were associated with significantly higher sexual pleasure and satisfaction scores for solo sex events, with no difference between lubricant types.
Herbenick 2014^26^	USA: national	Data focused on adult women from a subset of the 2012 National Survey of Sexual Health and Behaviour 2012 NSSHB. Mean age: 46.8, age range: 18–91. Participants were primarily white, non-Hispanic (66.5%) and identified as heterosexual (93.6%).	Quantitative: cross-sectional study	1559	The most common reason why women first started using lubricant was to “make sex more comfortable” (42.9%, *n* = 438). For women ages 18–39, however, the most commonly endorsed reason was “for fun”. “To enhance foreplay” and “curiosity” were also commonly reported. The most common perception was that a lubricant “makes sex more comfortable” (85.1%, *n* = 839). Over half agreed that lubricant use during sexual activities “makes sex feel better” and “makes it easier to feel aroused.” Less than 15% of the participants perceived lubricants to be only for older people.
Hickey 2016^27^	Australia	38 women having a history of breast cancer, being sexually active with symptoms of vaginal dryness or pain during sexual activity, willingness to be randomised and try both products, willingness to keep a sexual activity diary, and having a normal Pap smear in the previous 2 years. Mean age = 53.1.	Quantitative: randomised, double-blind, crossover trial (given lubricants as the intervention)	38	The majority of women reported that lubricants improved sexual experience and they would continue to use them. Silicone-based lubricants were generally preferred over water-based lubricants.
Javanbakht 2010^28^	multi-country (107) [specific countries not specified: North America; Europe; Latin America/Caribbean; Asia; Oceania; Other]: internet	6124 men and women who reported anal intercourse (AI) in the past 6 months, from North America (70%). Male (93%) respondents were older than female respondents (7%), with 55% of men being aged 35 years and older compared with 31% of women.	Quantitative: cross-sectional study (Internet based survey)	6124	Reasons for not using lubricants during AI including that they used saliva or vaginal fluid instead, lack of lubricant availability, or a preference for dry sex. Almost all said that lubricant color/flavor/smell did not matter, or they preferred no color/flavor/smell. Dispensers with a “pop-up” lid or a pump were most preferred, followed by tubes, single-use packets, and containers with screw-top or snap-off lids.
Jones 2008^29^	Zambia: Lusaka	155 HIV seropositive males, sexually active. Mean age: 37 years (Range: 21–62). 50% were unemployed, 27% worked part time. Ethnic groups included Bemba (27%), Nsenga, Ngoni, Tumbuka (26%), Tonga (14%), Lozi (15%), Mambwe, Namwanga (8%), and other ethnic groups (10%).	Quantitative: randomised trial without control groups and with assessments at baseline, monthly over 6 months and at 12 months (given lubricants as the intervention)	155	After 2 months of trial use, product ratings based on product selection and stated preference indicated that participants preferences remained fairly evenly distributed between suppositories, high-viscosity gel, and low-viscosity gel. Participants identified “ease of use,” “comfort,” and “increasing sexual pleasure” as the most important factors in product preference; being “fun” to use and “being in control” were considered “least important” factors. Two thirds of those sampled reported that both they and their partners “liked” the lubricants.
Jozkowski 2013^30^	USA: Internet	Mean age = 32.69, age range = 18 - 68, median = 31.0. Participants were predominantly white (87.7%, *N* = 2095), heterosexual (88.2, *N* = 2121), and were married and living with their spouse (57.7%, *N* = 1395).	Quantitative: cross-sectional study	2451	Women reported positive perceptions of lubricant, with younger women (18–24 and 25–29) reporting less positive perceptions than older women (40–49). Most women liked sex to feel wet, reported their partners preferred sex to feel wet, and reported being most easily orgasmic when sex was wet. Negative perceptions were when lubricants were perceived as sticky.
Juraskova 2013^4^	Australia	Adult female in a sexual relationship; amenorrheaic for at least 6 months or were taking aromatase inhibitors; had undergone adjuvant chemotherapy; and reported current symptoms of vaginal dryness and dyspareunia. Mean age: 51. Mean age at breast cancer diagnosis = 47.	Quantitative: prospective pilot study; time-series study across four time points (weeks 0, 4, 12, and 26) (given olive oil for lubricant as the intervention)	25	Overall, 76% of women found olive oil as a lubricant useful and all participants (100%) would recommend the intervention as a whole (Replents vaginal moisturiser, pelvic floor muscles, and olive oil) to other women with breast cancer who have similar problems.
Lee 2017^31^	South Africa: Pretoria	81 male, mean age = 25.16, range 20–39. Most participants identified as gay. The sample also included biologically male participants who self-identified as “drag queen”, women or transgender.	Qualitative: in-depth interviews	81	Facilitators to condom and lubricant use included: access to free condoms, partner dynamics (distrust), and increased acceptability to openly carry condoms and lubricants. Barriers included sexual initiation, issues with accessibility and availability, being in the heat of the moment, alcohol and drug use, partner dynamics, namely partner distrust again, and group sex.
Montgomery 2009^32^	Zimbabwe, South Africa	2523 in the intervention arm, and 2522 in the control arm. Age distribution <=24: 38.4%, 25–34: 39.1%, >=35: 22.5%. 58.9% of the participants are married. Lifetime # of sexual partners, mean (range):2.24(1–30). Age at first sex, mean (range):18.04 (10–31).	Quantitative: randomised controlled trial with 3 arms (diaphragm, gel and condoms (intervention) arm, or condoms-only (control) arm.)	5023	Only 1% of participants mentioned problems with the gel, including perceived burning or itchiness, increased discharge or wetness, or partner not liking the feeling of the gel during sex.
Reece 2014^33^	USA: national	1510 adult US males, mean age = 46.13, age range: 18–89. 67.3% participants were white, non-Hispanic with a sizable minority of participants indicating that they were black, nonHispanic (10.7%), or Hispanic (15.2%). Most identified as heterosexual (93.6%).	Quantitative: cross-sectional study	1510	Reasons for lubricant use included to make sex more comfortable, for fun (especially men aged 18–49), curiosity (especially men aged 18–49), partner preference for lubricant, to make sex more pleasurable, to reduce discomfort/pain during sex, makes it easier to feel aroused, makes sex feel better, makes it easier to have an orgasm. 10% of participants perceived that lubricants are only for older people
Rojanapithayakorn 1995^34^	Thailand: Ratchaburi, Ban Pong District and Damnoen Saduak District of Ratchaburi Province	female sex workers mean age = 22, age range 14 - 34. The average length of time the participant had worked in sex entertainment establishments (SEs) was 2.2 years. The participants had an average of 3.4 clients per day.	Quantitative; three time points (weeks 0 (before lubricant)), 1 (after 1week of lubricant as the intervention), and 8 (follow up 8 weeks later)	week 0: 134; week 1: 111; week 8: 58	About 95% of those interviewed expressed interest in using the water-soluble lubricant on a regular basis, because it reduced the time clients needed to ejaculate, reduced vaginal pain and discomfort and reduced condom breakage. More than 70% said the majority of their clients found using lubricants made condom use more enjoyable.
Romijnders 2015^35^	Tanzania: Dar es Salaam and Tanga	300 MSM, median age: 23 (IQR: 21–28); employed (81.3%); 58.3% self-identified as gay or homosexual, while 36.0% as MSMW	Quantitative: cross-sectional study	300	Reasons for using lubricants included that it feels better during anal sex and that it prevents condom tears or lesions during anal sex. Reasons for not using lubricant included availability (looking for lubricants only as the need arises, difficult to find, expensive). Only 8.7% disliked using lubricant.
Sahin-Hodoglugil 2011^36^	Multi-country (2): South Africa: Durban, Johannesburg; Zimbabwe: Harare	Sexually active, 18–49-year-old, HIV-negative women from five clinics. Women in FGD (*n* = 105): age: 18–24 years = 36 (36.5%); 25–34 years = 44 (42.3%); 35–49 years = 22 (21.2%). Women whose male partners participated (*n* = 41): age: 18–24 years = 14 (34.2%); 25–34 years = 21 (51.2%); 35–49 years = 6 (14.6%).	Qualitative: focus group discussions and in-depth interviews women (14 FGD) and 41 male partners (7 FGD plus 10 IDI) (given lubricants as part of intervention)	105	The gel was very well accepted and easily used by participants. Benefits included being seen as a product that increased sexual pleasure and stimulation for women, and relief from vaginal dryness and pain during intercourse. Only two men reported dissatisfaction with the gel as they preferred a dry vagina. Gendered sexual norms meant men had control over when/how often to have sex and what methods to use, and male sexual satisfaction was a larger theme than women’s sexual satisfaction.
Sanders 2018^37^	USA: Jackson, Mississippi	173 women recruited from an STI clinic engaged in penile-vaginal sex within the past three months. Mean age = 27.16, median age = 24, age range = 18–63. Most women identified as Black/African American (85.9%), with 4.7% identifying as White and the remainder not indicating a racial identity.	Quantitative: cross-sectional study	173	The majority of women were willing to experiment with condoms and lubricant. Lubricants were generally found to increase sexual pleasure. Negative perceptions of lubricants included when the lubrication amount was not enough to last until sex ended or maintain sexual satisfaction, or that lubrication “turned them off”.
Schick 2015^38^	USA: national	145 lesbian – and bisexually identified women and most recent sexual partner was a female. Age 18–24: 15.2%; 25–29: 21.1%; 30–39: 15.2%; 40–49: 18.9%; 50–59: 24.6%; 60+: 5.0%. 57.0% White/Non-Hispanic; 22.0% Black/Non-Hispanic; 10.9% Hispanic. 75.1% lesbian/homosexual; 24.9% bisexual.	Quantitative: cross-sectional study	145	Reasons for lubricant use included to make sex more comfortable, self or partner did not produce enough natural lubrication, reduce pain/discomfort during sex, increase pleasure during sex, and improve ability to orgasm/time to achieve orgasm/quality of orgasm. 65% agreed or strongly agreed that lubricant use improved their ability to orgasm, time to achieve orgasm, and quality of orgasm.
Steiner 1994^39^	USA: North Carolina (Raleigh, Durham and Chapel Hill)	268 couples, median age is slightly over 30 years (females: 31 years, males: 32 years) with a high level of formal education (median: females: 14.5 years, males: 15 years), predominately Caucasian (female: 85%, males: 84%).	Quantitative: pre-post study (Given lubricant as part of intervention)	536: 268 couples	Couples preferred the water-based lubricant over the oil-based lubricant (*p* < 0.001). When no additional lubricant was compared to the oil-based lubricant, couples preferred no additional lubricant (*p* < 0.001). When the comparison was between no additional lubricant and the water-based lubricant, they preferred the water-based lubricant (*p* < 0.001).
Sutton 2012^40^	Canada: internet	122 adult women. Dyspareunia group (*n* = 61), self-reported pain during or after penetrative intercourse, at least 50% of the time, for a 6-month duration, mean age = 29.85, 77% heterosexual; Control group (*n* = 61) reported no history or current chronic dyspareunia, mean age = 30.42, 77% heterosexual.	Quantitative: cross-sectional study	122 (61 control group, 61 dyspareunia group)	Lubricants were used to prevent or reduce pain, especially for women with dyspareunia. Reasons for not using lubricant were that it was not perceived as needed. Lubricants were used for masturbation or at the beginning of foreplay. Participants preferred water-based lubricants versus one with flavor, or lubricants with a tingling or warming sensation.

As described in WHO guideline on self-care interventions for health and well-being^47^

Support for lubricant use ranged from 55-100% across studies, as assessed with a wide range of measures across populations and settings, including whether participants would be “willing to experiment”, “would use again”, “would use on a regular basis”, “would recommend to others”, “acceptable”, and “liked very much”.

In three studies that compared water-based lubricant to either no lubricant or an oil-based lubricant, individuals generally preferred water-based lubricants.^25,39,40^ One study found that participants preferred odourless and tasteless lubricants, while another found that lubricant taste or smell did not matter, or participants preferred lubricants without flavour, colour, or smell.^22,28^

Reasons why individuals liked lubricants or would choose to use them ranged widely, and included comfort, reduced dryness/pain/discomfort, increased pleasure (for themselves or their partners), their partner’s preference, ease of orgasm (e.g. ability to orgasm, time needed to orgasm, quality of orgasm), preference for sex to feel more wet, more fun, curiosity, enhanced foreplay, clean, fast, easy insertion, reduced risk of tearing the vulva/vagina/anus, easier to feel aroused, increased readiness for sex, reciprocity, reduced chance of condoms drying out/breaking, and making condom use more enjoyable.

Reasons why individuals disliked lubricants or would choose not to use them also ranged widely, and included that lubricants were perceived as sticky, slippery, wet, messy, runny, gooey, burning, itchy, or leaky (nuisance); that lubricants were expensive, unavailable, or inaccessible; that individuals were not prepared when “in the heat of the moment” to quickly try using it, or that lubricant use interrupted sexual interaction; that individuals or their partners preferred dry sex or preferred to use non-commercial products (e.g. saliva, pre-seminal fluid) instead; or that participants perceived that lubricants were “only for older people”, or that they did not think they needed to use lubricant.

We identified no studies on values and preferences of health workers.

### Cost review

No studies presented primary data examining cost-effectiveness, cost-utility, or cost–benefit for lubricants.

## Discussion

Improving sexual health and well-being is an important but often neglected element of the WHO’s reproductive health strategy. Our systematic literature review highlights the limited evidence for this relatively low-cost and simple intervention. In our effectiveness review, we identified just one RCT that looked at sexual pleasure or well-being, finding that lubricants increased female sexual well-being. Though limited, this evidence does suggest that lubricants can be an important part of improving sexual health and well-being. However, more research is needed. In particular, sexual pleasure and well-being can only be measured subjectively and may be subject to self-report bias. This makes research challenging, particularly as it is difficult to blind participants to lubricant use. However, these are critical outcomes to measure when taking a positive approach to sexual health.

Our review found limited data on the association between lubricant use and STIs/HIV, except for one RCT showing that women who used lubricants were not more likely to acquire HPV. This finding is encouraging, particularly given the long-standing negative reverberations of evidence two decades ago that spermicides containing nonoxynol-9 did not protect against HIV infection and may even have increased HIV risk among women using these products frequently.^41^ While there has been substantial interest in lubricant use to reduce the risk of condom breakage and thus reduce HIV/STI risk,^42^ our review did not identify studies in this area. It is possible that many HIV trials provide condoms and lubricant to both study arms and thus do not provide comparative data on lubricant use versus no lubricant use, or that they measure outcomes such as condom slippage or breakage which were not in our *a priori* list of outcomes. It is also possible that our search string, which focused on studies with lubricant terms in the title or abstract, did not catch these trials.

Evidence on pain came from only cross-sectional studies and was of very low certainty for our questions of interest. One observational study showed lubricant use was associated with an increased proportion of gay and bisexual male participants reporting pain during receptive intercourse and no difference during insertive intercourse, but a reduced degree of pain in both types of intercourse. While this finding may raise concern, we interpret this as reverse causality in a cross-sectional survey. Participants who reported using lubricant during their last partnered event were asked to indicate their reasons for using lubricant; the most highly endorsed statement (89.3%) was that lubricant reduced their pain/discomfort. Men who experienced pain during receptive intercourse were more likely to use lubricants, but then experienced a reduced degree of pain, most likely because of the lubricant. A second observational study showed lubricant use was associated with better outcomes of vaginal dryness and dyspareunia for female breast cancer survivors. This study used olive oil as the lubricant of choice, along with pelvic floor muscle relaxation exercises and vaginal moisturiser, so the experience of participants and effectiveness of the regimen may be different from studies using other types of water- or petroleum-based lubricants. Although limited, these findings are overall encouraging for the impact of lubricants on pain and vaginal dryness.

While we identified 21 studies on values and preferences, these covered a range of country settings, methods, and population groups; additional evidence for specific populations and from lower-income settings is still needed. In particular, our findings on reasons why many individuals do not like lubricant may help product developers identify ways to improve current lubricant selection.

Although we identified no cost studies meeting our inclusion criteria, lubricants are available in many settings globally,^43^ with prices within the range of other over-the-counter sexual and reproductive health products. Currently, while the global lubricants market is dominated by North America (with approximately 37% of market share in 2019), the Asia Pacific region is the fastest-growing market.^44,45^ In addition, lubricants are also provided by many national HIV programmes in high-prevalence settings,^46^ and are thus within the realm of something that could feasibly be provided by many national health care services. However, further costing and cost-effectiveness research on lubricants for the outcomes we examined is needed.

A strength of this review is our inclusion of a range of research designs and our focus not only on effectiveness data, but also on values and preferences and cost data. We also conducted a comprehensive search and inclusion process, including multiple databases, secondary screening, and manual searches, with no exclusions based on language, location, or publication date. Furthermore, we followed best practices in systematic data extraction in duplicate. Limitations of our review include the focus on commercial or commonly available lubricants, excluding data on bodily fluids commonly used as lubricants (e.g. saliva, pre-seminal fluid), vaginal moisturisers, and microbicide gels. Given the range of body fluids, food products, and other items used as lubricants across settings, further study of non-commercial lubricant use is warranted. Further, our focus on peer-reviewed scientific articles may have limited inclusion of findings from market research or other relevant grey literature. As sexual health is a sensitive and private topic in many settings, findings may have been subject to social desirability bias, or individuals may not have felt fully comfortable expressing their true values and preferences. Finally, as noted above, many of our outcomes of interest are only measurable using self-report and it is difficult to blind participants to lubricant use; these factors may have introduced bias into the results of included studies.

Based on findings from this review as well as other inputs and discussion among diverse stakeholders, the World Health Organization self-care guideline made the following recommendation: “WHO recommends making lubricants available for optional use during sexual activity, among sexually active individuals”.^47^

## Conclusion

Overall, this systematic review found that lubricants may offer an accessible means to improve sexual wellbeing. The current reporting gives insights into values and preferences of end users and potentially important outcomes, but not the strength of evidence to reach conclusions on effectiveness or cost-effectiveness. Furthermore, although lubricants are a globally available product, study populations have lacked diversity. To fully inform guidelines, there is a need for improved research and reporting. Furthermore, publication bias is likely to have contributed to the fact that published studies tend to suggest effectiveness. Improving access to and availability of quality lubricants may contribute to the goals of respecting, protecting, and realising the right to health, and improving sexual health and well-being.

## Supplementary Material

Supplementary Table A

Supplemental Material - Search Terms

## Data Availability

All data come from published articles. Extracted data are available on request to the corresponding author.
