# Supplementary material for: Lubricants for the promotion of sexual health and well-being: a systematic review
Source: Sex Reprod Health Matters. 2022 Mar 22;29(3):2044198. doi: 10.1080/26410397.2022.2044198 (PMC8942543; doi:10.1080/26410397.2022.2044198)
Supplement: Supplementary Table A [file ZRHM_A_2044198_SM2169.docx]

**Appendix B. Table of excluded studies**

| **Author Year** | **Exclusion Reason** |
| --- | --- |
| Alary M, Peeters M, Laga M, et al.. HIV infection in European female sex workers: epidemiological link with use of petroleum-based lubricants. European Working Group on HIV Infection in Female Prostitutes. AIDS. 1993 Mar;7(3):401-8. | Prevalence/associations of lubricant use (cross-sectional study that studied prevalence of petroleum-based lubricant) |
| Ayoola OO, Sekoni AO, Odeyemi KA. Transactional sex, condom and lubricant use among men who have sex with men in Lagos State, Nigeria. African journal of reproductive health. 2013;17(4):90-98. | Prevalence/associations of lubricant use (lubricant use is the outcome; prevalence of use associated with sociodemographic characteristics/sexual behavior) |
| Braunstein S, van de Wijgert J. Preferences and practices related to vaginal lubrication: implications for microbicide acceptability and clinical testing. Journal of women's health. 2005;14(5):424-433. | Background (literature review) |
| Butler LM, Osmond DH, Jones AG, Martin JN. Use of saliva as a lubricant in anal sexual practices among homosexual men. Journal of acquired immune deficiency syndromes. 2009;50(2):162-167. | Prevalence/associations of lubricant use |
| Calabrese SK, Rosenberger JG, Schick VR, Novak DS, Reece M. An Event-Level Comparison of Risk-Related Sexual Practices Between Black and Other-Race Men Who Have Sex with Men: Condoms, Semen, Lubricant, and Rectal Douching. AIDS Patient Care & STDs. 2013;27(2):77-84. | Prevalence/associations of lubricant use |
| Chow EPF, Cornelisse VJ, Read TRH, et al. Saliva use as a lubricant for anal sex is a risk factor for rectal gonorrhoea among men who have sex with men, a new public health message: a cross-sectional survey. Sexually transmitted infections. 2016;92(7):532-536. | Not following our lubricant definition (bodily fluid) |
| Cornelisse VJ, Fairley CK, Read TRH, et al. Associations Between Anorectal Chlamydia and Oroanal Sex or Saliva Use as a Lubricant for Anal Sex: A Cross-sectional Survey. Sexually transmitted diseases. 2018;45(8):506-510. | Not following our lubricant definition (bodily fluid) |
| Crowell TA, Baral SD, Schwartz S, et al. Time to change the paradigm: limited condom and lubricant use among Nigerian men who have sex with men and transgender women despite availability and counseling. Annals of epidemiology. 2019;31:11-19.e13. | Prevalence/associations of lubricant use |
| Gafos M, Mzimela M, Sukazi S, et al. Intravaginal insertion in KwaZulu-Natal: sexual practices and preferences in the context of microbicide gel use. Culture, health & sexuality. 2010;12(8):929-942. | Not following our lubricant definition (gel microbicide) |
| Gelfand MM, Wendman E. Treating vaginal dryness in breast cancer patients: results of applying a polycarbophil moisturizing gel. Journal of Women's Health. 1994;3(6):427-434. | Not following our lubricant definition |
| Gorbach PM, Feaster DJ, Pines HJ, et al. P3.137 Rectal lubricant use & incident STI infections at 9 US STD Clinics. Sexually transmitted infections. 2013;89. | Not peer-reviewed (conference abstract) |
| Hoffman S, Morrow KM, Mantell JE, Rosen RK, Carballo-Diéguez A, Gai F. Covert use, vaginal lubrication, and sexual pleasure: a qualitative study of urban U.S. Women in a vaginal microbicide clinical trial. Archives of sexual behavior. 2010;39(3):748-760. | Not following our lubricant definition |
| Joglekar N, Joshi S, Kakde M, et al. Acceptability of PRO2000 vaginal gel among HIV un-infected women in Pune, India. AIDS care. 2007;19(6):817-821. | Not following our lubricant definition |
| Juraskova I, Jarvis S, Mok K, et al. Helping women “overcome” sexual problems after breast cancer treatment: An intervention study. Supportive Care in Cancer. 2011;19(2):S331. | Not peer-reviewed (conference abstract) |
| Kinsler JJ, Galea JT, Peinado J, Segura P, Montano SM, Sánchez J. Lubricant use among men who have sex with men reporting receptive anal intercourse in Peru: implications for rectal microbicides as an HIV prevention strategy. International journal of STD & AIDS. 2010;21(8):567-572. | Not following our lubricant definition (HIV prevention microbicide gel) |
| Kiran D, Manjunath R, Aswin KK, Patil B, Mahabalaraju D. A study on risk factors associated with inconsistent condom and lubricant use among men who have sex with men in central Karnataka, India. The Australasian medical journal. 2011;4(10):469-473. | Prevalence/associations of lubricant use (cross sectional study collecting quantitative data on lubricant use, no comparison of outcomes for lubricant use, no mention of costs, nor values and preferences related to lubricant use) |
| Laurie C, El-Zein M, Tota J, et al. Lubricant Investigation in Men to Inhibit Transmission of HPV Infection (LIMIT-HPV): Design and methods for a randomised controlled trial. BMJ open. 2020;10(3). | Protocol for RCT / no findings published yet |
| Milford C, Beksinska M, Smit J, Deperthes B. Lubrication and Vaginal Sex: Lubricant Use and Preferences in General Population Women and Women at Risk of HIV. Archives of sexual behavior. 2020;49(6):2103-2116. | Background |
| Mitchell CM, Reed SD, Diem S, et al. Efficacy of vaginal estradiol or vaginal moisturizer vs placebo for treating postmenopausal vulvovaginal symptoms a randomized clinical trial. JAMA internal medicine. 2018;178(5):681-690. | Not following our lubricant definition (microbicide) |
| Mitchell CM, Guthrie KA, Larson J, et al. Sexual frequency and pain in a randomized clinical trial of vaginal estradiol tablets, moisturizer, and placebo in postmenopausal women. Menopause. 2019;26(8):816-822. | Not following our lubricant definition (microbicide) |
| Picha E. [Lubrication reduces difficulties during sexual intercourse after gynecological surgery]. Akusherstvo i ginekologiia. 2002;41 Suppl 1:33-35. | Full text unavailable |
| Pines HA, Gorbach PM, Reback CJ, Landovitz RJ, Mutchler MG, Mitsuyasu R. Commercial lubricant use among HIV-negative men who have sex with men in Los Angeles: implications for the development of rectal microbicides for HIV prevention. AIDS care. 2014;26(12):1609-1618. | Prevalence/associations of lubricant use (qualitative study without values and preference mentioned; prevalence of lubricants use) |
| Ramanathan S, Chakrapani V, Ramakrishnan L, et al. Factors Associated with Use of Latex Condom-Compatible Lubricants by Men Who Have Sex with Men in India: Implications for HIV Prevention. Journal of sexually transmitted diseases. 2013;2013:161085. | Prevalence/associations of lubricant use (cross-sectional study assessed factors (exposure to HIV program, age, education, marriage status, employment, self-identity, alcohol assumption, any STI, HIV infection) associated with exclusive use of latex-condom compatible lubricants (water-based lubricants) among MSM.) |
| Ramjee G, Gouws E, Andrews A, Myer L, Weber AE. The acceptability of a vaginal microbicide among South African men. International Family Planning Perspectives. 2001:164-170. | Not following our lubricant definition (microbicide) |
| Ramjee G, Morar NS, Braunstein S, Friedland B, Jones H, van de Wijgert J. Acceptability of Carraguard, a candidate microbicide and methyl cellulose placebo vaginal gels among HIV-positive women and men in Durban, South Africa. AIDS research and therapy. 2007;4:20. | Not following our lubricant definition (microbicide) |
| Rao A, Ewing W, Ketende S, et al. Correlates of Water-Based Lubricant Use Among Men Who Have Sex with Men in Blantyre, Malawi. AIDS research and human retroviruses. 2019;35(9):833-841. | Prevalence/associations of lubricant use |
| Reece M, Mark K, Herbenick D, Hensel DJ, Jawed-Wessel S, Dodge B. An event-level analysis of adding exogenous lubricant to condoms in a sample of men who have vaginal sex with women. The journal of sexual medicine. 2012;9(3):672-678. | Prevalence/associations of lubricant use |
| Roddy RE, Cordero M, Ryan KA, Figueroa J. A randomized controlled trial comparing nonoxynol-9 lubricated condoms with silicone lubricated condoms for prophylaxis. Sexually transmitted infections. 1998;74(2):116-119. | Not following our lubricant definition |
| Rolle CP, Bolton MD, Kelley CF. Use of a Prospective Sex Diary to Study Anal Lubricant and Enema Use Among High Risk Men Who Have Sex With Men-Implications for Human Immunodeficiency Virus Prevention. Sexually transmitted diseases. 2016;43(8):476-478. | Prevalence/associations of lubricant use |
| Rosen RK, Morrow KM, Carballo-Diéguez A, et al. Acceptability of tenofovir gel as a vaginal microbicide among women in a phase I trial: a mixed-methods study. Journal of women's health (2002). 2008;17(3):383-392. | Not following our lubricant definition (microbicide) |
| Shieh EC, Weld ED, Fuchs EJ, et al. Lubricant Provides Poor Rectal Mucosal HIV Coverage. AIDS research and human retroviruses. 2017;33(8):784-787. | Not following our lubricant definition |
| Tanner AE, Zimet G, Fortenberry JD, Reece M, Graham C, Murray M. Young women's use of a vaginal microbicide surrogate: the role of individual and contextual factors in acceptability and sexual pleasure. Journal of sex research. 2009;46(1):15-23. | Not following our lubricant definition (microbicide) |
| Thienkrua W, Todd CS, Chaikummao S, et al. Lubricant Use Among Men Who Have Sex With Men Reporting Anal Intercourse in Bangkok, Thailand: Impact of HIV Status and Implications for Prevention. Journal of homosexuality. 2016;63(4):507-521. | Prevalence/associations of lubricant use (cross-sectional survey conducted among a MSM cohort in Thailand to assess factors associated with consistent lubricant use) |
| Whitehead SJ, Kilmarx PH, Blanchard K, et al. Acceptability of Carraguard vaginal gel use among Thai couples. AIDS (London, England). 2006;20(17):2141-2148. | Not following our lubricant definition (HIV prevention microbicide gel) |
