# Supplementary material for: Lubricants for the promotion of sexual health and well-being: a systematic review
Source: Sex Reprod Health Matters. 2022 Mar 22;29(3):2044198. doi: 10.1080/26410397.2022.2044198 (PMC8942543; doi:10.1080/26410397.2022.2044198)
Supplement: Supplemental Material - Search Terms [file ZRHM_A_2044198_SM2163.docx]

**Lubricants for promotion of sexual health and wellbeing: systematic review protocol**

Appendix A. Search Strategy

**Pubmed**

("lubricant"[tiab] OR "lubricants"[tiab] OR "lubrication"[tiab] OR "Alpha-Keri"[tiab] OR "Aquagel"[tiab] OR "Astroglide"[tiab] OR "ConceivEase"[tiab] OR "Conceive Plus"[tiab] OR "egg whites"[tiab] OR "FemGlide"[tiab] OR "Glycerin"[tiab] OR "Good Clean Love"[tiab] OR "H-R Jelly"[tiab] OR "Keri lotion"[tiab] OR "KY Jelly"[tiab] OR "Lubifax"[tiab] OR "MoisturizeR"[tiab] OR "Ortho-gynol"[tiab] OR "petroleum jelly"[tiab] OR "pHisohex"[tiab] OR "Pjur"[tiab] OR "Pre-seed"[tiab] OR "Pre Vaginal Lubricant"[tiab] OR "Replens"[tiab] OR OR "Searle skin lotion"[tiab] OR "Silk"[tiab] OR "Surgilube"[tiab] OR "Vaseline"[tiab] OR "baby oil"[tiab])

AND

("sex"[tiab] OR "sexual activity"[tiab] OR "sexual satisfaction"[tiab] OR "sexual stimulation"[tiab] OR "sexual function"[tiab] OR "sexual performance"[tiab] OR "sexual health"[tiab] OR "sexual wellbeing"[tiab] OR "sexual pleasure"[tiab] OR "sexual enhancement"[tiab] OR "intercourse"[tiab] OR "coitus"[tiab] OR "coital"[tiab] OR "comfort"[tiab] OR "comfortable"[tiab] OR "discomfort"[tiab] OR "uncomfortable"[tiab] OR "pleasure"[tiab] OR "reduce pain"[tiab] OR "pain reduction"[tiab] OR "friction"[tiab] OR "penetration"[tiab] OR "orgasm"[tiab] OR "arousal"[tiab] OR "fun"[tiab] OR "play"[tiab] OR "foreplay"[tiab] OR "intercourse"[tiab] OR "vaginal wetness"[tiab] OR dyspareunia [tiab])

**CINAHL**

AB ("lubricant" OR "lubricants" OR "lubrication" OR "Alpha-Keri" OR "Aquagel" OR "Astroglide" OR "ConceivEase" OR "Conceive Plus" OR "egg whites" OR "FemGlide" OR "Glycerin" OR "Good Clean Love" OR "H-R Jelly" OR "Keri lotion" OR "KY Jelly" OR "Lubifax" OR "MoisturizeR" OR "Ortho-gynol" OR "petroleum jelly" OR "pHisohex" OR "Pjur" OR "Pre-seed" OR "Pre Vaginal Lubricant" OR "Replens" OR OR "Searle skin lotion" OR "Silk" OR "Surgilube" OR "Vaseline" OR "baby oil")

AND

AB ("sex" OR "sexual activity" OR "sexual satisfaction" OR "sexual stimulation" OR "sexual function" OR "sexual performance" OR "sexual health" OR "sexual wellbeing" OR "sexual pleasure" OR "sexual enhancement" OR "intercourse" OR "coitus" OR "coital" OR "comfort" OR "comfortable" OR "discomfort" OR "uncomfortable" OR "pleasure" OR "reduce pain" OR "pain reduction" OR "friction" OR "penetration" OR "orgasm" OR "arousal" OR "fun" OR "play" OR "foreplay" OR "intercourse" OR "vaginal wetness" OR dyspareunia)

**LILACS**

"lubricant" [palavras]

**Embase**

TI,AB,KW ('lubricant' OR 'lubricants' OR 'lubrication' OR 'Alpha-Keri' OR 'Aquagel' OR 'Astroglide' OR 'ConceivEase' OR 'Conceive Plus' OR 'egg whites' OR 'FemGlide' OR 'Glycerin' OR 'Good Clean Love' OR 'H-R Jelly' OR 'Keri lotion' OR 'KY Jelly' OR 'Lubifax' OR 'MoisturizeR' OR 'Ortho-gynol' OR 'petroleum jelly' OR 'pHisohex' OR 'Pjur' OR 'Pre-seed' OR 'Pre Vaginal Lubricant' OR 'Replens' OR OR 'Searle skin lotion' OR 'Silk' OR 'Surgilube' OR 'Vaseline' OR 'baby oil')

AND

TI,AB,KW ('sex' OR 'sexual activity' OR 'sexual satisfaction' OR 'sexual stimulation' OR 'sexual function' OR 'sexual performance' OR 'sexual health' OR 'sexual wellbeing' OR 'sexual pleasure' OR 'sexual enhancement' OR 'intercourse' OR 'coitus' OR 'coital' OR 'comfort' OR 'comfortable' OR 'discomfort' OR 'uncomfortable' OR 'pleasure' OR 'reduce pain' OR 'pain reduction' OR 'friction' OR 'penetration' OR 'orgasm' OR 'arousal' OR 'fun' OR 'play' OR 'foreplay' OR 'intercourse' OR 'vaginal wetness' OR dyspareunia)

**Handsearch Parameters**

**Google Scholar**

allintitle: ((lubricant OR lubricants OR lubrication) (sex OR sexual OR intercourse OR coitus OR coital OR foreplay OR pleasure OR orgasm OR dysfunction OR arousal OR dyspareunia))

Other parameter: no patents

**ClinicalTrials. gov**

Conditions: lubricating agents OR Inadequate Lubrication OR Sexual Dysfunction OR Coitus OR Physiological OR Vaginal Diseases OR Human Papillomavirus Infection OR Contraception OR Dyspareunia OR Vaginal Atrophy OR Gonorrhea OR Chlamydia OR Trichomoniasis OR Syphilis OR Infection OR Human Immunodeficiency Virus OR HIV Infections OR Female Dry Genital Mucosa OR HIV

Other search term: lubricant

Other parameter: Results available

**WHO International Clinical Trials Registry Platform, Pan-African Clinical Trials Registry, and Australian New Zealand Clinical Trials Registry**

("lubricat*" OR "Alpha-Keri" OR "Aquagel" OR "Astroglide" OR "ConceivEase" OR "Conceive Plus" OR "egg whites" OR "FemGlide" OR "Glycerin" OR "Good Clean Love" OR "H-R Jelly" OR "Keri lotion" OR "KY Jelly" OR "Lubifax" OR "MoisturizeR" OR "Ortho-gynol" OR "petroleum jelly" OR "pHisohex" OR "Pjur" OR "Pre-seed" OR "Pre Vaginal Lubricant" OR "Replens" OR "Searle skin lotion" OR "Silk" OR "Surgilube" OR "Vaseline" OR "baby oil") AND ("sex*" OR "sexual activity" OR "sexual satisfaction" OR "sexual stimulation" OR "sexual function" OR "sexual performance" OR "sexual health" OR "sexual wellbeing" OR "sexual pleasure" OR "sexual enhancement" OR "intercourse" OR "coit*" OR "comfort*" OR "discomfort" OR "uncomfortable" OR "pleasure" OR "reduce pain" OR "pain reduction" OR "friction" OR "penetration" OR "orgasm" OR "arousal" OR "fun" OR "play" OR "foreplay" OR "intercourse" OR "vaginal wetness" OR “dyspareunia”)

**Other parameter**: with results only

**Cochrane Library**

(("lubricant" OR "lubricants" OR "lubrication" OR "Alpha-Keri" OR "Aquagel" OR "Astroglide" OR "ConceivEase" OR "Conceive Plus" OR "egg whites" OR "FemGlide" OR "Glycerin" OR "Good Clean Love" OR "H-R Jelly" OR "Keri lotion" OR "KY Jelly" OR "Lubifax" OR "MoisturizeR" OR "Ortho-gynol" OR "petroleum jelly" OR "pHisohex" OR "Pjur" OR "Pre-seed" OR "Pre Vaginal Lubricant" OR "Replens" OR "Searle skin lotion" OR "Silk" OR "Surgilube" OR "Vaseline" OR "baby oil")):ti AND (sex):ti,ab,kw" (Word variations have been searched)
